# Vitamin B12 Regulates the Transcriptional, Metabolic, and Epigenetic Programing in Human Ileal Epithelial Cells

**DOI:** 10.3390/nu14142825

**Published:** 2022-07-09

**Authors:** Yong Ge, Mojgan Zadeh, Mansour Mohamadzadeh

**Affiliations:** Department of Medicine, Division of Gastroenterology & Nutrition, University of Texas Health, San Antonio, TX 78229, USA; gey@uthscsa.edu (Y.G.); zadehm1@uthscsa.edu (M.Z.)

**Keywords:** vitamin B12, ileal epithelial cells, mitochondrial metabolism, DNA methylation, cell proliferation

## Abstract

Vitamin B12 (VB12) is a micronutrient that is essential for DNA synthesis and cellular energy production. We recently demonstrated that VB12 oral supplementation coordinates ileal epithelial cells (iECs) and gut microbiota functions to resist pathogen colonization in mice, but it remains unclear whether VB12 directly modulates the cellular homeostasis of iECs derived from humans. Here, we integrated transcriptomic, metabolomic, and epigenomic analyses to identify VB12-dependent molecular and metabolic pathways in human iEC microtissue cultures. RNA sequencing (RNA-seq) revealed that VB12 notably activated genes involved in fatty acid metabolism and epithelial cell proliferation while suppressing inflammatory responses in human iECs. Untargeted metabolite profiling demonstrated that VB12 facilitated the biosynthesis of amino acids and methyl groups, particularly *S*-adenosylmethionine (SAM), and supported the function of the mitochondrial carnitine shuttle and TCA cycle. Further, genome-wide DNA methylation analysis illuminated a critical role of VB12 in sustaining cellular methylation programs, leading to differential CpG methylation of genes associated with intestinal barrier function and cell proliferation. Together, these findings suggest an essential involvement of VB12 in directing the fatty acid and mitochondrial metabolisms and reconfiguring the epigenome of human iECs to potentially support cellular oxygen utilization and cell proliferation.

## 1. Introduction

VB12 is a cobalt-containing micronutrient that is synthesized by a subset of bacteria and archaea [1], and its production is regulated by bacterial riboswitches [2]. Although some microbes of mammalian guts possess the capacity to synthesize VB12, the receptor (cubilin) needed for VB12 absorption is expressed only by epithelial cells of the ileum, wherein microbial abundancy and diversity are low [3]. Diets, particularly animal products, comprise the primary natural source of VB12 in humans. It is for this reason that vegetarians, and especially vegans, are more commonly found to possess low serum VB12 levels [4]. VB12 delivery from foods to tissues involves multiple transport proteins and their cell surface receptors. VB12 is first released from foods in the upper gastrointestinal tract with the help of gastric acid, pepsin, and haptocorrin and then binds to intrinsic factor (IF) secreted by parietal cells of stomach to form a VB12-IF complex, which reaches the ileum and is absorbed by ileal epithelial cells (iECs) through binding to cubilin [5]. Upon receptor-mediated endocytosis and the subsequent lysosomal degradation of IF, VB12 is released by iECs and enters the circulation, where it is bound to transcobalamin and taken up by CD320-expressing cells [6]. 

In humans, VB12 acts as a co-factor for two enzymes: cytosolic methionine synthase (MS) and mitochondrial methylmalonyl-CoA mutase (MCM). MS catalyzes the first step in the biosynthesis of methionine, transferring the methyl group from methyltetrahydrofolate to homocysteine [7]. The impairment of MS activity due to VB12 deficiency leads to the accumulation of homocysteine (termed hyperhomocysteinemia), thereby contributing to the formation of reactive oxygen species (ROS), which is a risk factor for the development of cardiovascular and neurological diseases [8,9]. Diminished MS activity also reduces the levels of methionine, a precursor for the universal methyl donor *S*-adenosylmethionine (SAM) that is essential for supporting the cellular DNA methylation process [10]. Several studies report potential associations between VB12 deficiency and DNA hypomethylation [11,12,13], whereas other studies document no correlation or even a reverse association [14,15]. Thus, the involvement of VB12 in DNA methylation is still controversial and requires further investigations. 

Additionally, VB12, functioning as a crucial cofactor for MCM, converts methylmalonyl-CoA to succinyl-CoA, a process that takes place in the mitochondria. Succinyl-CoA not only participates in the TCA cycle but can also serve as the precursor for the biosynthesis of heme, which is critically involved in erythropoiesis and cellular respiration [16,17]. Accordingly, VB12 deficiency has been implicated in pernicious anemia and hematological manifestations [18]. Further, VB12 deficiency also results in the accumulation of serum methylmalonic acid (termed methylmalonic aciduria) [19], which may inhibit the mitochondrial electron transport chain [20]. In a recent prospective study of human adults, circulating levels of methylmalonic acid have been significantly associated with cardiovascular mortality, potentially due to induced mitochondrial dysfunction and oxidative stress [21]. Recent studies also show that VB12 deficiency may disturb the mitochondrial homeostasis in *Caenorhabditis elegans* [22,23]. However, the underlying cellular mechanisms linking VB12 to mitochondrial regulation remain obscure. 

We recently demonstrated that VB12 dietary deficiency reduces the mitochondrial gene expression and carnitine shuttle activity of iECs in mice [24]. VB12 deficiency also decreases the production of gut microbiota-associated fatty acids that potentiate the peroxisome proliferator-activated receptor (PPAR) signaling pathway in iECs during intestinal *Salmonella* infection, leading to the diminished function of fatty acid β-oxidation. Compromised mitochondrial activity correlates with reduced oxygen consumption by iECs, resulting in the expansion of intestinal oxygen-dependent *Salmonella* in VB12-deficient mice. Additionally, VB12 deficiency impairs the production of propionate and butyrate in the distal gut, which may directly inhibit the expression of *Salmonella* virulence genes that are important for epithelial invasion. Despite the importance of VB12 supplementation in regulating the iECs and microbiota functions in mice, the direct causative relationship between VB12 and iEC–mitochondrial metabolism is lacking. Further, there is sparse evidence on direct associations between VB12 and iEC function in humans. Using a 3-dimensional (3D) microtissue model, we comprehensively examined the impacts of VB12 on the transcriptomic, metabolomic, and epigenetic programming of human iECs in vitro. Our results correlatively suggest that VB12 not only facilitates the fatty acid and mitochondrial metabolisms of human iECs, but also sustains DNA methylation programs that are essential for epithelial cell proliferation and function.

## 2. Materials and Methods

### 2.1. Human iECs and VB12 Treatment

The organotypic 3D human iEC microtissues, which closely mimic native human small intestine tissue, were developed using cells derived from the ileum region of a 19-year-old female donor [25]. Tissue specimens were collected from the brain-dead donor after receiving informed consent from the next of kin. The microtissues were obtained from commercial sources (MatTek Corporation, Ashland, MA, US) and cultured using proprietary serum-free medium (MatTek Corporation, Ashland, MA, US) supplemented with VB12 (cyanocobalamin; 500 nM) [26] or PBS for two weeks. The tissues were maintained under submerged conditions with medium contacting both the apical and basolateral sides of the tissue. When the cells reached confluence, the medium was removed from the apical side and the cells were exposed to an air–liquid interface to stimulate tissue maturation and to form the organotypic 3D tissue model [25]. Samples were shipped on dry ice and kept at −80 °C upon receipt.

The transcriptomic raw data of murine iECs were downloaded from NCBI (accession number PRJNA750489). These iECs were isolated from VB12-deficient mice gavaged with VB12 or PBS for 1, 5, and 8 weeks, as previously described [24], and were compared to human iECs to analyze conserved gene homologs in both humans and mice.

### 2.2. RNA-Seq Analysis

RNA was isolated from human iECs (~5 × 10^4^ cells/sample) using the RNeasy Plus Micro kit (Qiagen, Germantown, MD, USA). cDNA libraries were constructed using the SMART-seq protocol as previously described [27] and sequenced on an Illumina NovaSeq 6000 system at the University of Florida ICBR NextGen DNA Sequencing Core Facility. The obtained raw reads were aligned to human (GRCh38.83) reference genomes using STAR v2.7.5c. Normalized transcripts per million reads (TPM) were generated using RSEM v1.3.3. Subsequently, DESeq2 was used to determine significantly expressed genes (DEGs) based on the criteria (TPM > 1, false discovery rate < 0.05, fold change > 1.5). Normalized counts were log2-transformed, and a principal component analysis (PCA) was performed using R package Factoextra. Heat maps of TPM expression were generated using Pheatmap in R. Gene set enrichment analysis (GSEA) was performed using DAVID (https://david.ncifcrf.gov/, accessed on 12 June 2022). Network analysis using STRING version 11.5 was performed for the top-50 up and down DEGs.

### 2.3. Quantitative Real-Time PCR (qPCR)

To validate DEGs identified by RNA-Seq, cDNA samples synthesized with SMART-Seq HT Kit (Takara, San Jose, CA, USA) to construct RNA-seq libraries were used. qPCR was performed with the PowerUp SYBR Green Master Mix (Thermo Fisher Scientific, Waltham, MA, USA) on a QuantStudio 6 Pro real-time PCR system (Thermo Fisher Scientific, Waltham, MA, USA). The relative quantification (2^−ΔCt^) was used to determine the expression level of the target genes normalized *GAPDH* using primers listed in Supplemental Table S1.

### 2.4. Metabolomic Analysis

Human iECs (about 5 × 10^5^ cells) were processed for metabolite extraction as described previously [28] with pre-normalization to the sample protein content (300 µg/mL). The samples were run on a Thermo Fisher Q-Exactive Obitrap mass spectrometer with a Dionex UHPLC and autosampler at the Southeast Center for Integrated Metabolomics (www.secim.ufl.edu, accessed on 12 June 2022). The samples were analyzed in both positive and negative ionization with a mass resolution of 35,000 at *m*/*z* 200 as separate injections. Feature alignment and curation were performed by MZmine through an automated routine developed in-house. Missing values were imputed with half minimum intensity of the dataset. After normalizing to total ion chromatogram, the intensities were tested for group significance using a two-tailed unpaired *t*-test. Metabolites were identified by comparison to a metabolomic library of purified standards. The metabolic pathway analysis was performed using *Mummichog* [29] with default parameters. The pathways represented by at least two significant metabolites in a positive or negative mode are presented. 

### 2.5. Genome-Wide Methylation Analysis

Genomic DNA was isolated from human iECs cultured with VB12 or PBS (~2 × 10^5^ cells/sample) using the Quick-DNA Microprep Plus Kit (Zymo Research, Irvine, CA, USA). A bisulfite-converted DNA library was constructed using the NEBNext Enzymatic Methyl-seq Kit and sequenced on an Illumina NovaSeq 6000 platform. 

Raw sequencing reads were first trimmed by Trim Galore v0.6.6 to remove poor-quality nucleotides. High-quality reads were then mapped to human (GRCh38.83) reference genomes using Bismark v0.22.3, yielding more than 15× the average coverage across the genome. The bisulfite conversion rates were determined by a spike-in of fully methylated lambda DNA. Differentially methylated regions (DMRs) were identified using Bioconductor package DSS. We first performed statistical tests of differentially methylated loci using DSS and the results were used to detect DMRs using the CallDMR function in DSS with a *P* threshold for calling DMR set at 0.01. The minimal length for a DMR was set to 50 bp with a minimal delta of 0.1 spanning at least 5 CpG sites. The genomic annotation of DMRs was annotated using BEDTools v2.29.2 with gene annotations downloaded from the UCSC Table Browser. Promoters were defined as 2 kb upstream and 200 bp downstream of the transcription start sites. The enhancer database was downloaded from GeneHancer. The genomic location of DMRs was assigned in the following order: promoter, enhancer, coding exon, 5′ UTR, 3′ UTR, intron, and intergenic regions. Each DMR was assigned in the order listed above and each DMR was assigned to only one category.

### 2.6. Statistical Analysis

The statistical differences between the VB12 and PBS control groups were analyzed by performing a two-tailed unpaired *t*-test using GraphPad Prism v9.1.2. Data are presented as the mean ± SD. *p* < 0.05 were considered as significant: * *p* < 0.05, ** *p* < 0.01, *** *p* < 0.001.

## 3. Results

### 3.1. Transcriptomic Programming of Human iECs Cultured in the Presence of VB12

**Figure 1:**
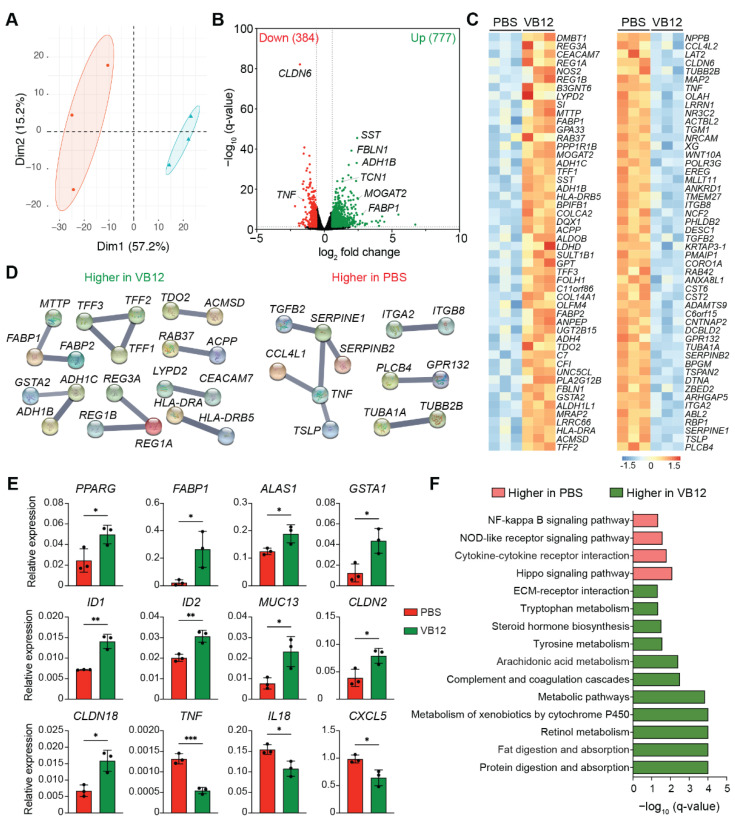
VB12-dependent regulation of transcriptomic responses in human iECs. The organotypic 3D human small intestine microtissues were cultured in the presence of VB12 or PBS (n = 3 samples/group) using cells obtained from the ileal region of a 19-year-old female donor. Two weeks later, cells were collected for RNA-seq analysis. (**A**) PCA plot of transcriptomes of human iECs cultured in the presence of VB12 versus PBS. (**B**) Volcano plot of differentially expressed genes (DEGs; FDR < 0.05, fold change > 1.5) between VB12 and PBS groups. The number of upregulated (green) and downregulated (red) DEGs in the VB12 group is shown in parentheses. (**C**) Heat map showing top-50 DEGs. (**D**) STRING analysis depicting the networks of the top-50 DEGs. (**E**) qPCR validation of representative DEGs. (**F**) KEGG pathways of DEGs enriched in the VB12 versus PBS group. Bar graphs show mean ± SD. * *p* < 0.05, ** *p* < 0.01, *** *p* < 0.001; two-tailed unpaired *t*-test (**E**).

To elaborate on the VB12-dependent regulation of human iECs, we analyzed the transcriptomes of the 3D microtissues reconstructed using human ileal fibroblasts and enterocytes [25] cultured in the presence of VB12 versus PBS. Compared to PBS controls, human iECs cultured with VB12 demonstrated an evidently different transcriptome (Figure 1A), with a total of 1161 differentially expressed genes (DEGs) (9.76% of total expressed genes), among which 66.9% were upregulated through the addition of VB12 (Figure 1B). The search tool for recurring instances of neighboring genes (STRING) analysis depicted interactive networks of the top-50 DEGs (Figure 1C,D). The upregulated clusters included genes associated with chylomicron formation (*MTTP*, *FABP1*, and *FABP2*), which are important for fatty acid absorption and cellular transport as well as trefoil factors (*TFF1*, *TFF2*, and *TFF3*) (Figure 1D)—a family of mucin-associated secretory molecules that can maintain and restore gastrointestinal homeostasis [30]. We consistently observed an enhanced expression of mucin-encoding genes (e.g., *MUC13*) and tight junction proteins (*CLDN2* and *CLDN18*) in the VB12 group (Figure 1E), thereby indicating VB12’s potential role in modulating intestinal barrier function. Notably, *PPARG*, a key transcriptional regulator of fatty acid β-oxidation [31], was also increased in human iECs by VB12 (Figure 1E). Additionally, members of a regenerating gene family (*REG1A*, *REG1B*, and *REG3A*) exhibited anti-apoptotic and pro-proliferative properties [32], and transcriptional factors (*ID1* and *ID2*) that regulate cell proliferation were both found with elevated levels in the VB12 group when compared to PBS controls (Figure 1D,E). In contrast, the top downregulated DEGs, including *TNF*, *CCL4L1*, *SERPINE1*, and *SERPINE2*, were primarily associated with an inflammatory response.

Kyoto Encyclopedia of Genes and Genomes (KEGG) and Gene Ontology (GO) enrichment analyses further demonstrated that VB12-cultured human iECs were enriched with pathways associated with intestinal digestion and absorption, oxidation-reduction process, and epithelial cell differentiation, whereas control iECs exhibited a proinflammatory phenotype with enriched pathways related to cytokine–cytokine receptor interaction and NF-κB signaling (Figure 1F and Supplemental Figure S1A). Additionally, arachidonic acid metabolism and the associated genes, particularly phospholipases A2 (*PLA2G2A*, *PLA2G4F*, *PLA2G10*, and *PLA2G12B*) (Supplemental Figure S1B), which catalyze the hydrolysis of phospholipids to generate free fatty acids [33], were also upregulated in human iECs cultured with VB12. These iECs were also enriched with transcripts of the mitochondrial carnitine palmitoyltransferase 1A (*CPT1A*) and carnitine/acylcarnitine translocase (*SLC25A20*), both of which are involved in carnitine shuttling, a process that is important for transporting fatty acids into mitochondria, thereby highlighting the critical role of VB12 in modulating mitochondrial fatty acid metabolism of human iECs. 

### 3.2. Transcriptomic Correlation between Human and Murine iECs

**Figure 2:**
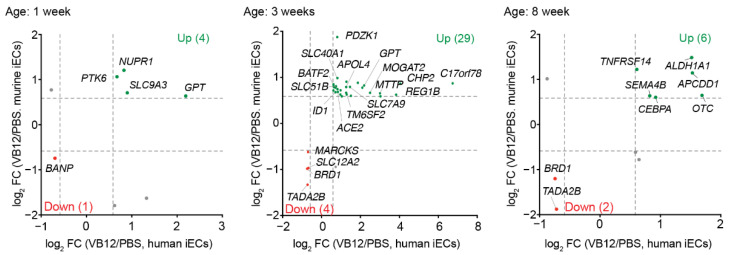
Comparison of human and mouse iEC-enriched genes. Green dots indicate genes upregulated in both human iECs cultured with VB12 and iECs isolated from VB12-gavaged mice of different ages (1, 5, and 8 weeks old), compared to their PBS counterparts. Red dots indicate genes downregulated in the VB12 group of both human and mouse iECs. Dashed lines indicate fold changes of 1.5. log_2_ FC, log_2_ (fold change).

Using a mouse model for VB12 deficiency, we recently demonstrated that VB12 oral supplementation leads to the transcriptomic reprogramming of murine iECs, which varies with mouse ages [24]. To identify enriched gene homologs in the iECs of both humans and mice, we next compared the 1161 genes enriched in humans with iECs derived from mice of different ages (1, 5, and 8 weeks old) (Figure 2). Despite the evolutionary distance and differences in tissue origin, 33 human iEC-enriched gene homologs were found in iECs isolated from mice that were gavaged with VB12 versus PBS for 5 weeks (20.4% of total DEGs), most of which were upregulated by VB12 (29 out of 33). The genes with conserved iEC expression included those encoding components for intestinal lipid absorption (*MTTP*, *APOL4*, *MOGAT2*, and *TM6SF2*); transporters for irons, amino acids, and bile acids (*SLC40A1*, *SLC7A9*, and *SLC51B*); and transcriptional factors (*ID1* and *BATF2*) that regulate cell proliferation (Figure 2). Interestingly, VB12 increased the expression of the SARS-CoV-2 receptor angiotensin-converting enzyme 2 (*ACE2*) in both murine and human iECs. Among the four downregulated gene homologs, transcriptional adaptor 2B (*TADA2B*) is a member of the histone acetyltransferase complex [34], bromodomain-containing 1 (*BRD1*) is involved in histone acetylation and erythropoiesis [35], and myristoylated alanine-rich C kinase substrate (*MARCKS*) may inhibit epithelial mucin secretion [36]. Furthermore, genes associated with lipid metabolism (*ALDH1A1* and *OTC*), epithelial differentiation (*APCDD1* and *CEBPA*), and histone modifications (*BRD1* and *TADA2B*) were also shared between human iECs and the iECs derived from 8-week-old mice. At the age of 1 week, only a few murine gene homologs were shared with human iECs (Figure 2), which might be due to the shortened time length of VB12 exposure. These results thus suggest that VB12 supports the fatty acid metabolism and cell proliferation of both human and murine iECs.

### 3.3. Metabolic Regulation of Human iECs by VB12

**Figure 3:**
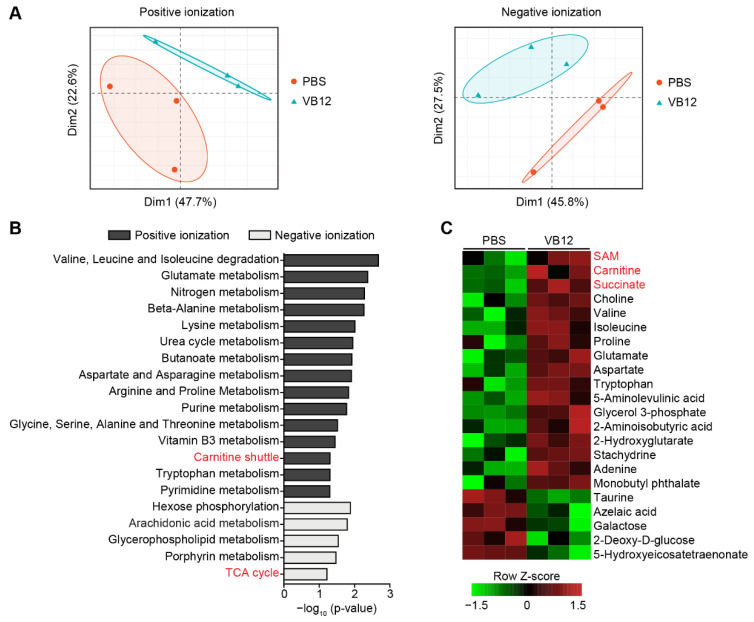
Metabolic programming of human iECs by VB12. (**A**) PCA plots of metabolite features identified by positive and negative ionization in human iECs cultured with VB12 or PBS for 2 weeks (n = 3 samples/group). (**B**) Significant metabolic pathway between VB12- and PBS-cultured iECs. (**C**) Heatmap showing differentially enriched metabolites. SAM, *S*-adenosylmethionine.

A liquid chromatography–mass spectrometry-based metabolomic analysis demonstrated that the addition of VB12 notably led to the metabolic reprogramming of human iECs (Figure 3A), which were primarily defined by altered pathways associated with amino acid metabolisms (Figure 3B). The mitochondrial carnitine shuttle and TCA cycle were also significantly modified by VB12. Accordingly, human iECs experiencing VB12 were enriched with amino acids, including valine, isoleucine, proline, glutamate, aspartate, and tryptophan (Figure 3C). These iECs also had elevated levels of mitochondrial metabolites that are involved in the carnitine shuttle (carnitine), TCA cycle (succinate and 2-hydroxyglutarate), and heme biosynthesis (5-aminolevulinic acid). Additionally, choline and glycerol 3-phosphate related to lipid metabolism, stachydrine exerting anti-inflammatory properties [37], as well as SAM supporting cellular methylation programs were all detected with increased levels in human iECs cultured with VB12. 

In contrast, 5-hydroxyeicosatetraenonate, an arachidonic acid metabolite reported to increase intestinal epithelial paracellular permeability [38], and 2-deoxy-D-glucose, a glucose analogue that effectively inhibits glucose metabolism, were reduced in the VB12 culture (Figure 3C). The intracellular taurine, which is increased in the intestinal epithelial cell line upon exposure to hypertonic stress [39], was also decreased by VB12. Interestingly, levels of azelaic acid, which regulates the inflammatory response by inducing PPARγ activation [40], were diminished by VB12 culturing. Together, these results indicate an important role of VB12 in modulating cellular metabolic homeostasis, which influences the mitochondrial function and proliferation of human iECs.

### 3.4. DNA Methylation Profile in the Presence of VB12

**Figure 4:**
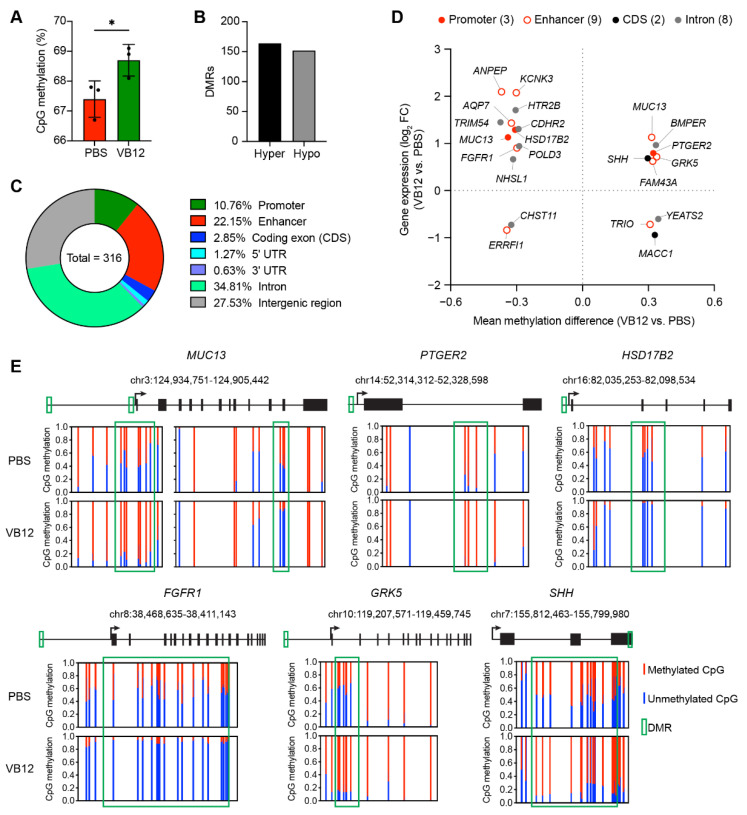
VB12-mediated alterations in genome-wide DNA methylation profiles of human iECs. (**A**) Global CpG methylation ratios of human iECs cultured with VB12 or PBS for 2 weeks (n = 3 samples/group). (**B**) Summary of the number of differential methylation regions (DMRs) between VB12- and PBS-cultured human iECs from WGBS analysis. (**C**) Donut chart representative of the DMR genomic distribution. UTR, untranslated region. (**D**) Comparison of DEGs and DMRs between VB12 and PBS groups. The number of DMRs within indicated locations of the DEGs is shown in parentheses. (**E**) Normalized plots of CpG methylation at sites surrounding or within DMRs of genes associated with barrier function (*MUC13*) and cell proliferation (*PTGER2*, *FGFR1*, *GRK5* and *SHH*) obtained from WGBS analysis. The red and blue lines depict methylated and unmethylated CpG sites, respectively. * *p* < 0.05; two-tailed unpaired *t*-test (**A**).

Having shown that VB12 sustained the levels of SAM, the methyl donor critically involved in cellular methylation programs, we investigated whether transcriptional and metabolic programming was accompanied by additional epigenetic modifications in human iECs. Thus, we generated whole-genome bisulfite sequencing (WGBS) profiles of three biological replicates of human iECs cultured with VB12 or PBS (Supplemental Table S2). Data demonstrated that global CpG methylation levels were significantly higher in the VB12 group as compared to PBS controls (Figure 4A). In contrast to CpG methylation, methylation in the CH sequence context was low, and there was no significant difference in the CHH or CHG methylation levels (Supplemental Table S2). Further analysis defined differential methylation regions (DMRs), including 164 hypermethylated regions and 152 hypomethylated regions in VB12-cultured iECs relative to PBS controls (Figure 4B), both of which were significantly enriched in promoters and enhancers (Figure 4C). 

Next, we correlated these DMRs with genes that were differentially expressed between the VB12 and PBS groups to identify epigenetic events that were potentially implicated in the regulation of gene expression. Here, 21 DEGs were detected with altered methylation within their promoters, enhancers, coding DNA sequence (CDS), or introns (Figure 4D). DNA methylation at the promoter region generally silences gene expression [41]. Accordingly, we observed that hypomethylated promoters of *MUC13* and *HSD17B2* were associated with increased gene expression. Additionally, a second DMR with enhanced DNA methylation was detected in the enhancer element of *MUC13* (Figure 4D,E), highlighting the role of VB12-dependent DNA methylation in controlling *MUC13* expression.

Contributing to intestinal barrier function, a GO analysis of the 21 DEGs further demonstrated that differential DNA methylation was primarily associated with a changed expression of genes involved in cell proliferation (*PTGER2*, *SHH*, *CHST11*, *FGFR1*, *GRK5*, and *HTR2B*). DEGs related to DNA replication (*POLD3*), microvilli packing (*CDHR2*), and epithelial cell digestive function (*AQP7* and *ANPEP*) also had DMRs within their gene regulatory elements (Figure 4D). Moreover, the hypermethylated intron of *BMPER*, a protective regulator of inflammation [42], is correlated with increased gene expression, whereas the hypomethylated enhancer of *ERRFI1* (also known as gene 33), which can be induced by proinflammatory cytokines [43], was associated with a reduced gene expression in VB12-cultured iECs (Figure 4D). Altogether, these results suggest that VB12-induced epigenetic programming may be important for supporting cell proliferation and function as well as restricting the inflammatory response in human iECs.

### 3.5. Transcriptional, Metabolic and Epigenetic Integration

**Figure 5:**
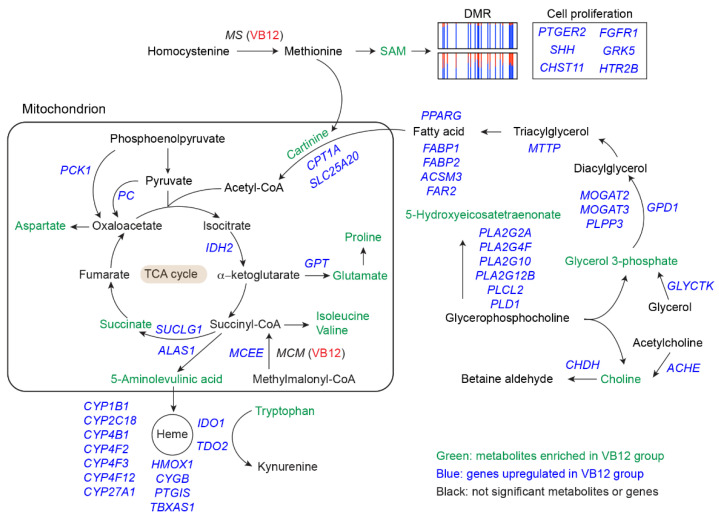
Summary model based on transcriptomic, metabolomic, and epigenomic integration. As a crucial co-factor for MCM, VB12 supports the generation of succinyl-CoA, which can participate in the TCA cycle and serve as the precursor for heme biosynthesis, resulting in the activation of heme enzymes. VB12 also facilitates fatty acid metabolism, which potentially generates free fatty acids that can be transported into mitochondria to fuel the TCA cycle through the carnitine shuttle. Further, VB12 sustains the MS activity to enhance the biosynthesis of carnitine and SAM, which supports the carnitine shuttle and influences epigenetic programs of genes involved in cell proliferation. An elevated TCA cycle also contributes to the production of amino acids that are essential for cell proliferation.

Correlating the obtained transcriptomic, metabolomic, and epigenomic results reveals an integrated role of VB12 in supporting lipid metabolism and mitochondrial TCA cycle, thereby leading to sustained mitochondrial respiration and cell proliferation of human iECs (Figure 5). In this process, VB12 serves as a co-factor for MS for the generation of methionine, facilitating the biosynthesis of SAM, which notably impacts the methylation and expression of genes involved in cell proliferation (e.g., *PTGER2*, *SHH*, and *CHST11*). Methionine is also one of the two amino acids required for carnitine biosynthesis and thus promotes the function of the carnitine shuttle that is critical for transporting cytosolic fatty acids into mitochondria. Meanwhile, VB12 activates genes associated with fatty acid transport (e.g., *FABP1*, *FABP2*, and *MTTP*) and metabolic activities (e.g., phospholipases and *PPARG*), potentially generating fatty acids for mitochondrial oxidation. Additionally, VB12 functions as a co-factor for mitochondrial MCM, which is responsible for the production of succinyl-CoA, and not only participates in the TCA cycle but also serves as the precursor for the heme biosynthesis that affects the heme-dependent enzyme activities, such as *IDO1* and *TDO2*, involved in tryptophan metabolism. A sustained TCA cycle also favors the biosynthesis of amino acids (e.g., aspartate), which are necessary to meet the metabolic demand for the cellular proliferation of human iECs. 

## 4. Discussion

VB12 is an essential micronutrient that is primarily acquired through dietary intake. The primary risk factor for humans developing a VB12 deficiency is a lack of production of IF produced by gastric parietal cells, which is needed for the intestinal absorption of VB12 [18]. Other contributing factors include dietary restriction (e.g., malnutrition), *Helicobacter pylori* infection, and intestinal bacterial overgrowth [44,45]. Accumulative studies have shown that VB12 deficiency has been linked to various well-described reversible hematological disorders as well as often reversible neurological disorders [46]. However, the mechanisms by which VB12 regulates the cellular programs of intestinal epithelial cells to resist disease progression remain poorly understood. We have recently demonstrated that VB12, when orally given to mice, modifies the gut microbiota and iEC functions, resulting in the control of intestinal *Salmonella* infection [24]. Here, we conducted a complementary study to directly assess the impact of VB12 on the cellular machinery of human iECs in culture.

Small intestinal epithelium serves as a critical gatekeeper that controls dietary lipid absorption and systemic distribution. Small intestinal epithelial cells, including iECs, absorb lipids and distribute them to different intracellular lipid pools. Some of these lipids are used by iECs for fatty acid oxidation to support cellular metabolism or for phospholipid biosynthesis to form cell membranes, while others may be esterified and packed into chylomicron lipoproteins for secretion into lymphatic vessels for systemic distribution [47]. We observed not only the increased expression of transcripts involved in chylomicron assembly, particularly *FABP1*, *FABP2*, and *MTTP*, but also the enhancement of genes that are critically associated with fatty acid oxidation, including *PPARG*. We [24] and others [48,49] have revealed that the impairment of PPAR signaling is a key feature of VB12 deficiency in vivo. Here, we found that the expression of PPARG was also compromised in human iEC cultures when VB12 was absent, once again illuminating the significance of PPAR in relation to VB12-mediated cell signaling. Additionally, correlating the transcriptomic data from both human and murine iECs demonstrated that iECs had an enhanced expression of genes related to fatty acid metabolism after exposure to VB12. Consistent with our observation of murine iECs, human iECs cultured with VB12 were also enriched with genes (*CPT1A* and *SLC25A20*) and metabolites (carnitine) of the carnitine shuttle, a critical mechanism for the mitochondrial utilization of fatty acids. These observations, along with an enrichment of TCA cycle-related genes (e.g., *IDH2* and *SUCLG1*) and metabolites (succinate and glutamate) in VB12-cultured human iECs, support the notion that VB12 regulates the mitochondrial metabolism in both human and murine iECs. 

Compared to iECs of VB12-deficient mice, iECs derived from mice gavaged with VB12 show the increased transcription of mitochondrially encoded genes (e.g., *mt-Nd1*), which are crucial for mitochondrial respiration during both steady-state and STm infection [24]. No such mitochondrial gene activation was seen in human iECs cultured with VB12. This might be due to the absence of functional microbiota or the associated metabolites [50]. For example, nicotinamide is the precursor of nicotinamide adenine dinucleotide (NAD), which is essential for multiple metabolic pathways, including the mitochondrial electron transport chain [51]. This metabolite is significantly higher in the intestinal lumen of VB12-gavaged mice as compared to VB12-deficient mice [24]. Butyrate, which is also enriched in VB12-gavaged mice, can enter the TCA cycle and reduce NAD^+^ to NADPH in colonocytes [50]. While these metabolites have been implicated in mitochondrial respiration, their relationship with the expression of mitochondrial genes still requires further investigations.

As a crucial co-factor for MCM, VB12 supports the generation of succinyl-CoA, which may favor the TCA cycle and heme biosynthesis. The mitochondrial 5-aminolevulinate synthase 1 (*ALAS1*) is the rate-limiting enzyme in heme biosynthesis that converts succinyl-CoA to 5-aminolevulinic acid (5-ALA), the common precursor for all naturally occurring tetrapyrroles [52]. Both the expression of *ALAS1* and the levels of 5-ALA were significantly higher in human iECs cultured with VB12 than those cultured with PBS, indicating VB12′s role in regulating heme biosynthesis. Heme is involved in oxygen homeostasis by sensing and using molecular oxygen [53]. Enzymes such as catalases, peroxidases, and cytochrome P450 rely on heme as essential co-factors, and thus heme deficiency leads to severe metabolic disorders called porphyrias in humans [54]. Heme degradation is mediated by heme oxygenase, including heme oxygenase 1 (*HMOX1*), which has been reported to activate mitochondrial biogenesis and mediate anti-inflammatory effects [55]. The expression of *HMOX1* may be induced by intracellular hypoxia in VB12-cultured human iECs [56], as these cells were enriched with gene sets associated with the oxidation–reduction process. Additionally, the expression of cytoglobin (*CYGB*), which is induced upon hypoxia to protect against oxidative stress [57], and NADPH oxidase 1 (*NOX1*), which robustly consumes oxygen in intestinal epithelial cells [58,59], were both activated by VB12. These results indicate that VB12 may promote oxygenation utilization in human iECs, a phenomenon reminiscent of our in vivo mouse study that showed that VB12 enhances iEC capability to consume oxygen, which resulted in the maintenance of a hypoxic gut environment that is critical for controlling aerobic *Salmonella* infection [24].

Numerous studies have documented the associations between serum vitamin B12 status and DNA methylation [11,12,13,14,15,60], which is generally believed to be the result of an altered methionine metabolism. However, there is a lack of evidence to support the direct involvement of VB12 in epigenetic modifications. Here, we clearly demonstrated that the addition of VB12 to human iEC cultures led to the enrichment of methyl donor SAM and accordingly increased the genome-wide CpG methylation, resulting in differential methylations of genes involved in intestinal barrier function (*MUC13*) and cell proliferation (e.g., *SHH*). Additionally, choline, which may provide an alternative source of methyl groups [61], was also enriched in human iECs cultured with VB12, suggesting that VB12 supports the cellular methyl pools in human iECs. 

## 5. Conclusion

Our results uncovered the critical role of VB12 in regulating the cellular transcriptional, metabolic, and epigenetic programs, which result in elevated mitochondrial function and sustained cell proliferation of human iECs. These novel properties of VB12 may advance the usage of this micronutrient in clinical settings to maximize its contribution to human health.

## Data Availability

All data associated with this study are present in the paper or the Supplemental materials. The transcriptomic and epigenomic raw FastQ files of human iECs have been deposited into NCBI BioProject under the accession number PRJNA750453. The transcriptomic FastQ files of murine iECs are available under the accession number PRJNA750489.

## Supplementary Materials

The following supporting information can be downloaded at: https://www.mdpi.com/article/10.3390/nu14142825/s1. Table S1: Primers used for qPCR; Table S2: Summary of enzymatic Methyl-seq data processing; Figure S1: Transcriptional regulation of human iECs by VB12.
