# Supplementary material for: Vitamin B12 Regulates the Transcriptional, Metabolic, and Epigenetic Programing in Human Ileal Epithelial Cells
Source: Nutrients. 2022 Jul 9;14(14):2825. doi: 10.3390/nu14142825 (PMC9321803; doi:10.3390/nu14142825)
Supplement: Supplementary file 1 [file nutrients-14-02825-s001.zip › Figure S1.pdf]

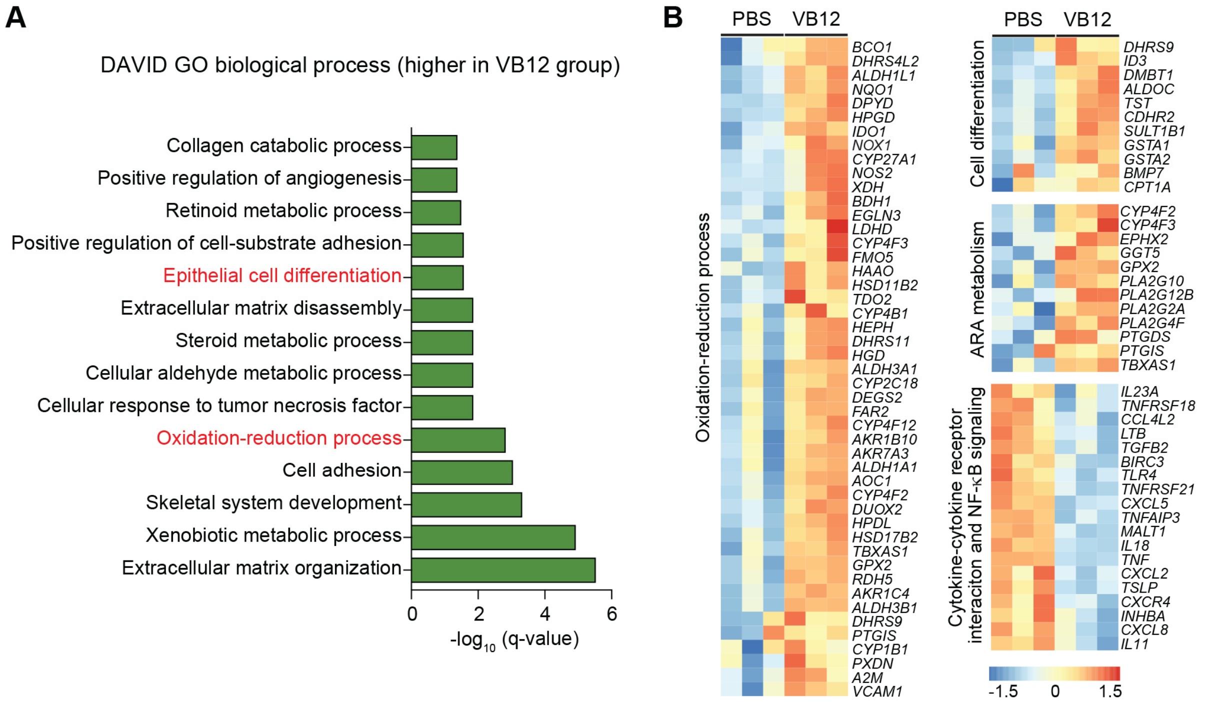

Figure S1. Transcriptional regulation of human iECs by VB12. **(A)** Significant DAIVD Gene Ontology (GO) pathways in VB12-cultured human iECs. **(B)** Heat maps of DEGs associated with indicated pathways. ARA, arachidonic acid.
